# Approximate resolution convolution function for fitting a dispersion gap measured on a triple-axis spectrometer

**DOI:** 10.1107/S1600576726002049

**Published:** 2026-04-22

**Authors:** Emma Y. Lenander, Silas B. Schack, Kim Lefmann, Henrik M. Rønnow

**Affiliations:** a Niels Bohr Institute, University of Copenhagen, DK-2100 Copenhagen Ø, Denmark; b Institute of Physics, École Polytechnique Fédérale de Lausanne (EPFL), CH-1015 Lausanne, Switzerland; Technical University of Denmark, Denmark

**Keywords:** analytical convolution functions, gap size, spin waves, triple-axis spectrometers, resolution functions

## Abstract

We present an analytical convoluted gap function for fitting dispersion gaps measured on a triple-axis spectrometer (TAS). At the gap, the instrumental resolution skews the signal, producing a high-energy tail that complicates fitting. Our function assumes an instrumental *Q* resolution with two equal wide directions and one narrow direction (typical of focused TAS instruments) and a parabolic dispersion at the gap, which is exact for quadratic and accurate for linear dispersions if the resolution is moderate. We demonstrate that our function outperforms previous methods of fitting a gap by giving a better fit and more accurate gap determination. Here, the antiferromagnetically gapped material MnF_2_ is simulated in a double-focusing TAS instrument. We also tested our function on experimental data on MnF_2_ from a TAS-like instrument, where we reproduced the gap size from previous accurate measurements determined experimentally. The function is simple to implement and converges reliably, and we recommend its use for future gap fitting on TAS data.

## Abstract 2

An analytical convoluted gap function is presented for fitting either a linear or a quadratic dispersion gap in a constant-*Q* cut measured with a focused triple-axis spectrometer. It outperforms previous methods of fitting a gap, without requiring a full resolution convolution.

## 1. Introduction

Neutron spectroscopy is a very powerful technique for investigating magnetic and lattice dynamics in materials as a function of momentum and energy transfer. The neutron scattering cross section is proportional to the spatial and temporal Fourier transform of the respective atomic displace­ment and spin–spin correlation functions (Boothroyd, 2020 ▸), embodied in the dynamic structure factor *S*(**Q**, ω). In many cases, the ground state spontaneously breaks the continuous symmetry of a system, resulting in gapless symmetry-restoring excitations (Goldstone modes). In crystals, the lattice dynamic is well described by phonons, and in magnetically ordered systems the primary magnetic excitations are spin waves. Phonons restore the broken translational symmetry of a crystal, and spin waves restore the rotational spin symmetry in Heisenberg and *XY* models. In both cases, these coherent collective excitations give rise to dispersive bands of momentum-dependent excitation energies with corresponding structure factors:
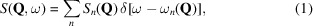
where ω_*n*_(**Q**) denotes the dispersion relation of the *n*th mode and *S*_*n*_(**Q**) its corresponding spectral weight (Shirane *et al.*, 2002 ▸). The δ function enforces energy conservation such that each branch contributes only at its characteristic excitation energy. The dispersion of the Goldstone modes close to zero energy can be linear (*e.g.* acoustic phonons and antiferromagnetic spin waves) or quadratic (*e.g.* ferromagnetic spin waves and triplon excitations). For a quadratic excitation, *S*_*n*_(**Q**) is constant, while for a linear excitation, *S*_*n*_(**Q**) ∝ 1/ω.

Various effects including anisotropy, pinning, incomplete softening or long-range interactions can gap these excitations. Detecting and quantifying such gaps (Δ) reveals some of the key information that can be extracted from spectroscopic investigations. Gapping of a quadratic Goldstone mode leads to the following generic dispersion: 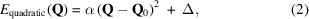
where α determines the curvature of the parabola and 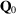 is where the dispersion has its minimum energy. Gapping of a linear dispersion leads to the following generic dispersion in the vicinity of the gap: 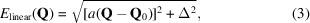
where *a* is the spin-wave velocity (the slope of the dispersion at **Q** values away from the gap region). We here assume that the spin-wave velocity is identical for all crystallographic directions. In the limit **Q** → **Q**_0_ [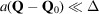] we perform a series expansion of equation (3), giving the parabolic form of equation (2) with α = *a*^2^/(2Δ) > 0.

In neutron spectroscopy, the measured signal is not an ideal δ function but is broadened by the finite resolution of the instrument. This broadening is described by the resolution function *G*(**Q** − **Q**′, ω − ω′), which represents the spectrometer’s response in both momentum and energy space. The resolution function describes how a true signal at (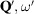) is broadened by the finite resolution of the instrument when measured at (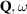). The respective *Q* and *E* resolutions determine how finely the momentum and energy dependence of the excitation can be resolved: limited *Q* resolution and *E* resolution will both broaden and smear the dispersion in the experimental data.

**Figure 1:**
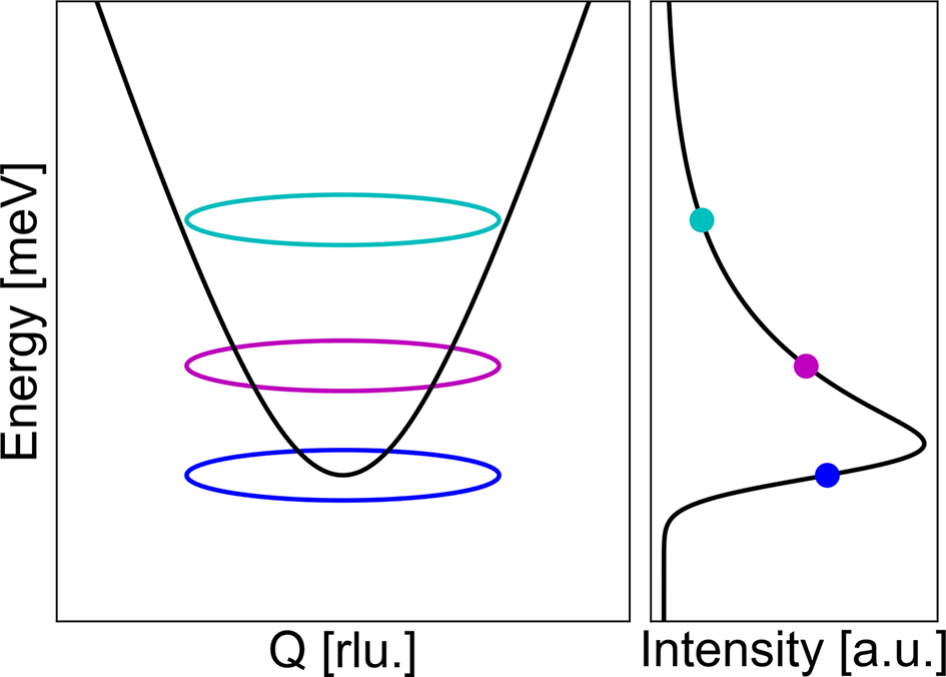
(Left) The linear dispersion in equation (3), energy transfer as a function of **Q** (black line). The instrumental resolution at the gapped **Q** position is drawn as coloured ellipses at varying energy transfers. (Right) The intensity in a constant-*Q* cut at the gapped position, illustrating how the high-energy tail above the spin gap appears from the instrumental resolution.

The observed intensity is thus given by the convolution of the intrinsic scattering function *S*(**Q**, ω) with the instrumental resolution: 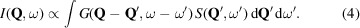
This convolution broadens, and in some cases skews, the theoretical δ-function peaks in *S*(**Q**, ω), producing the experimentally observed intensity distribution (Fig. 1 ▸). To obtain the exact cross section, one must also include the fundamental constants, the dipole factor, and the ratio between the incident and final neutron wavevectors (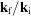).

**Figure 2:**
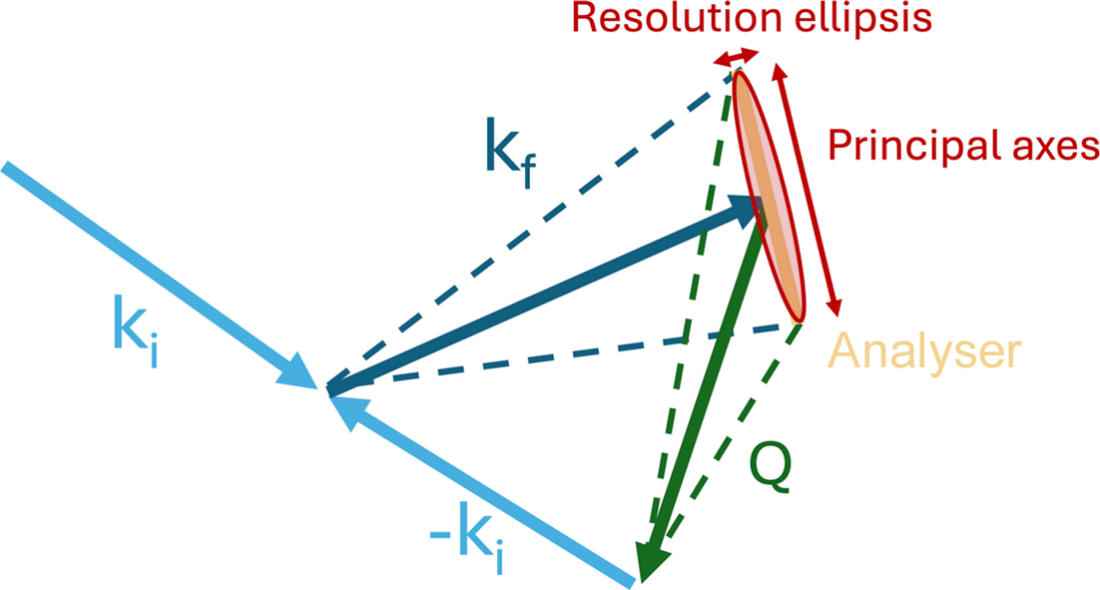
The two-dimensional part of the *Q* resolution ellipsoid for a triple-axis spectrometer, indicated in red. The scattering vector **Q** = **k**_f_ − **k**_i_, with the size of the analyser, determines the 3D *Q* resolution, which can be split into three components: two d*Q* principal axes in the scattering plane, one narrow and one wide, and d*Q*_vert_, which is the resolution out of the scattering plane (wide, and not drawn). The instrument also has an energy resolution which needs to be taken into account.

The shape and width of the resolution function depend on several factors, including **k**_i_ and **k**_f_, the mosaic spreads of the monochromator and analyser crystals, the collimation setup, the distances between instrument components, the scattering angles, and the wavelength spread of the neutrons (Fig. 2 ▸). Thus, a full resolution calculation requires detailed knowledge of the experimental configuration, and information about the sample properties may also be necessary to model the scattering volume properly. Analytical derivations of the resolution function typically assume that each component of the neutron optics exhibits a Gaussian transmission or reflection profile with a certain variance (Shirane *et al.*, 2002 ▸). Under this assumption, the convolution of all contributions yields a four-dimensional Gaussian resolution function *G*(**Q**, ω). By virtue of the central limit theorem, the final resolution ellipsoid is well approximated by a Gaussian distribution even when the individual components are not strictly Gaussian. Consequently, resolution functions obtained, for example, from Monte Carlo ray-tracing simulations can be accurately fitted with a Gaussian profile without requiring that each underlying optical component itself follow a Gaussian distribution.

In practice, the form and anisotropy of the resolution function vary between spectrometer types. One typically measures dynamics on a time-of-flight (TOF) spectrometer or a triple-axis spectrometer (TAS). A pixellated TOF spectrometer often has relatively fine *Q* resolution, which can be tuned after the experiment by balancing bin width versus statistics. In contrast, a focusing TAS generally has wider *Q* resolution in two of three directions, one of these being the vertical direction. For a TAS, the instrument resolution can be determined analytically using the Cooper–Nathans (Cooper & Nathans, 1967 ▸) and Popovici (Popovici, 1975 ▸) formalisms to compute the resolution matrix **M** under the assumption that all uncertainties have Gaussian distributions and linear approximations around the nominal instrument settings. In this framework, the resolution function can be written as 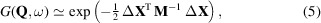
where Δ**X** = (Δ**Q**, Δω). The resolution matrix can be computed numerically by Monte Carlo integration, as implemented in software packages such as *Takin* (Weber, 2023 ▸), or through ray-tracing simulations using *McStas* (Lefmann & Nielsen, 1999 ▸; Lefmann *et al.*, 2000 ▸; Willendrup & Lefmann, 2020 ▸). One can also determine it experimentally by scanning single-crystal or powder Bragg peaks, and we present a guide to this in Appendix *A*[App appa].

**Figure 3:**
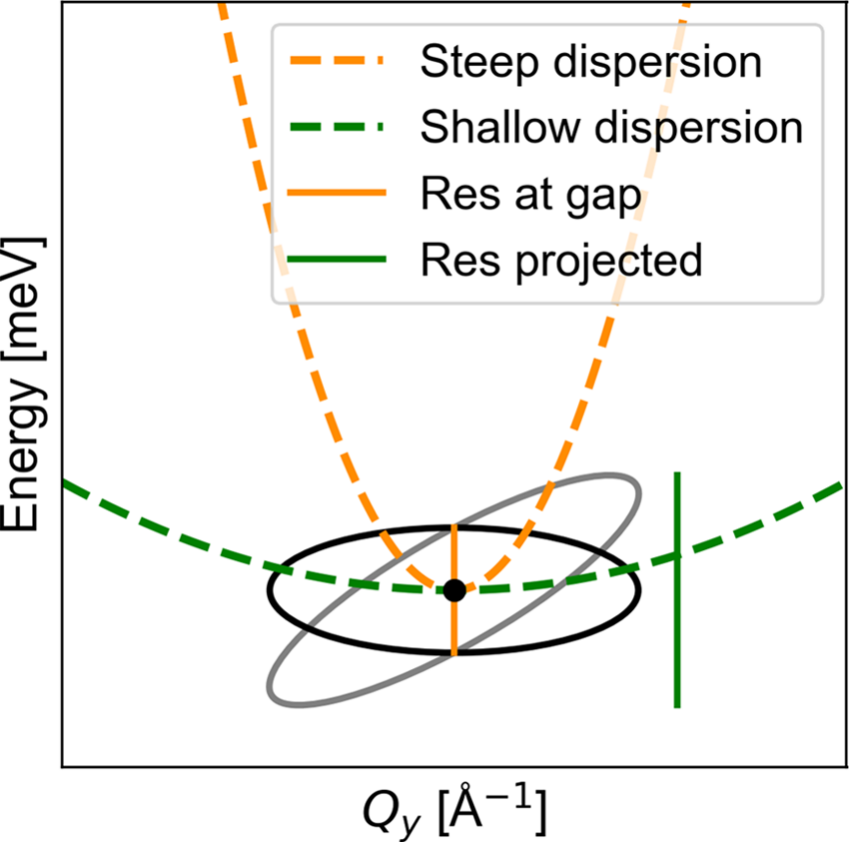
The intrinsic instrumental energy resolution at a specific **Q**, shown in orange, and the projected effective resolution, shown in green. For a shallow dispersion, one should use the projected resolution (green), while for a steep dispersion (orange), the energy resolution at a specific **Q** is more correct. For our approximation, we do not see the correlations between *Q* and *E* as in the real case (grey ellipsis), so we assume an ellipsis with no tilt (black) and with the energy resolution depending on the steepness of the dispersion.

In this article, we consider the case of an instrument with a broad *Q* resolution in two of the three reciprocal-space directions, representative of a focused TAS setup. The remaining direction, with the shortest principal axis of the ellipsoid, is approximated by a delta function, δ(*Q*_*x*_). Here, *x* is in general not aligned with the crystallographic axis, but this is unproblematic in our isotropic approximation. The other two principal axes are modelled by Gaussian resolution functions of equal width, denoted σ_*Q*_. The energy resolution is represented by a Gaussian of width σ_*E*_. The assumptions of the resolutions are that the *Q* resolution has the shape of a pancake and the energy resolution depends on the steepness of the dispersion. For a steep dispersion compared with the resolution ellipsis, indicated in orange in Fig. 3 ▸, one will use the intrinsic instrumental energy resolution at a specific **Q** (at the gap). For a shallow dispersion, one should use the effective projected energy resolution (green). Under these assumptions, the total resolution function can be approximated as 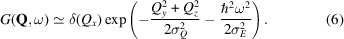
This is one of the approximations that we will make in order to derive an analytical function for the resolution convoluted scattering function.

To measure the gap of the dispersion with a TAS experimentally, one usually performs a constant-*Q* scan (or cut) at the bottom of the dispersion. Thus, the data consist of intensity [*I*(**Q**, ω)] as a function of energy transfer (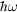). Taking the instrument resolution into account, the approximation that the dispersion follows a parabola at the gap [equation (3)] is valid for the linear excitation when the series expansion is valid or when the *Q* resolution is narrower than the sides of the parabola. If the energy variation of the linear dispersion and the instrument’s energy resolution are both small compared with the energy gap, and the former is within the *Q* resolution range, then 1/ω can be approximated as a constant, like the quadratic excitation. A gapped dispersion can, therefore, be described by equation (2) with *S*_*n*_(**Q**) being constant. This results in the scattering function in equation (1) being written as 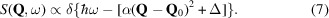
This is our second approximation, which will be used in order to derive the analytical function for the resolution convoluted scattering function.

Normally, to determine the gap size of a constant-*Q* scan, one will typically fit the dispersion with a Gaussian line shape and a constant or linear background. This works well away from the gap, but at the bottom of the dispersion, due to the instrument resolution, the scan tends to pick up signal from higher energies. This results in a tail at energies above the gap (Fig. 1 ▸) which is not captured in a Gaussian line shape. To obtain a more precise value for the gap position, one can use a function that captures the tail. A typical example of such a function is a Gaussian line shape for the low-energy part of the peak and a Voigt profile for the high-energy part of the peak (Appendix *B*[App appb]). The Voigt profile is a convolution of a Gaussian and a Lorentzian function; thus it has a Gaussian peak shape with a Lorentzian tail. This function has the potential to fit better, but it is not a physical description of the resolution and the function tends to overestimate the gap size.

In this article, we propose an approximate function to fit the cut at the gap to a skewed function: a convoluted gap model [equation (11)]. Here we use the two approximations mentioned above; that the gapped dispersion follows a parabola at the gap position and that the *Q* resolution has two equally wide directions and one vanishingly narrow direction, typical for a focusing TAS.

The function also includes the energy resolution and the spin-wave velocity of the gapped dispersion. By simulating an antiferromagnetically gapped material MnF_2_ in a double-focusing TAS instrument, we show that our function performs much better than the Gaussian line shape and the combined Gaussian–Voigt function. We have also tested our function on experimental data on MnF_2_ from the TAS-like instrument CAMEA (PSI, Villigen, Switzerland). Even in the case of a TAS-like instrument, we find that our function fits the data well and succeeds in finding the gap value.

## 2. Analytical resolution convoluted function for fitting a gap

Using the approximations presented in the previous section [equations (6) and (7)], we can write the intensity given by equation (4) as 
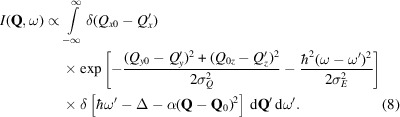
From this expression for the intensity, we present an analytical solution, the details of the derivation being described in Appendix *C*1. We find that the intensity is described as a convolution of the energy resolution with an asymmetric function *f*(ω) that includes the *q* part of the resolution. Since *f*(ω) only picks up intensity above the gap, 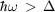, the function includes the Heaviside function, *H*: 
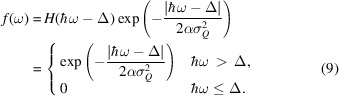
From this, the final intensity becomes 
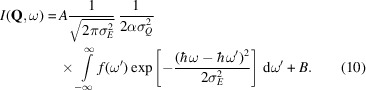
The Heaviside function in *f*(ω) restricts the domain of the integration to 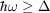, effectively changing the integration interval to [Δ, ∞). The Gaussian normalization constants are written explicitly, while all other experimental constants, *e.g.* the prefactor of *S*(**Q**, ω) and the intensity normalization of the instrument, are described by a normalization constant *A*. To account for the instrument background, a scalar *B* is used.

Equation (10) is to be determined numerically, which makes it more difficult in practice for a fitting routine to converge at the global minimum. Thus, we are interested in finding an analytical expression (Appendix *C*2). The Heaviside function in *f*(ω) in equation (9) ensures support only for 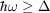, but the convolution itself yields a smooth non-zero tail for all 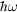 given by the error function (erfc). The analytical convoluted gap function is finally written by 
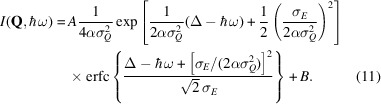
If the approximation of the *Q* resolution holds, the analytical function in equation (11) is exact for gapped quadratic dispersions like ferromagnetic spin waves. It is valid for gapped linear excitations, like phonons or antiferromagnetic spin waves, under the assumptions that the excitation at the gap follows a parabola and that the factor *S*_*n*_(**Q**) ∝ 1/ω can be approximated as a constant. For the linear excitation, one can replace α = *a*^2^/(2Δ). For more details on the assumptions, see Appendix *D*[App appd].

## 3. Simulated data: MnF_2_

To demonstrate the use of our expression for the analytical resolution convoluted gap function [equation (11)], we choose to study examples from the antiferromagnet MnF_2_. This material has been extensively studied as a model system in magnetism, particularly through neutron scattering. Its simple structure and well defined magnon dispersion make it a benchmark material for understanding antiferromagnetic spin dynamics (Yamani *et al.*, 2010 ▸).

MnF_2_ is a well known antiferromagnetic insulator that crystallizes in the tetragonal rutile structure (space group *P*4_2_/*mnm*). It has a Néel temperature of approximately 67 K, below which Mn^2+^ ions (*S* = 5/2) align antiparallel along the *c* axis (Erickson, 1953 ▸), forming a collinear antiferromagnetic order. Its magnon dispersion is gapped with an energy gap of approximately 1.082 meV at the zone centre. This was measured with a precision of 0.006 meV with an antiferromagnetic resonance (AFMR) experiment (Johnson & Nethercot, 1959 ▸).

AFMR probes the **Q** = 0 (uniform) magnon modes of an antiferromagnet by measuring their microwave or terahertz resonance frequencies. Since it directly measures these zero-momentum excitations, AFMR can determine the spin gap with exceptionally high energy precision, typically of the order of microelectronvolts. However, in many frustrated or anisotropic systems the true minimum of the magnon dispersion occurs at finite **Q**, and such modes are invisible to AFMR. Thus, MnF_2_ represents a case where AFMR is ideally suited, since the minimum of its magnon dispersion lies at **Q** = 0.

Inelastic neutron scattering on a thermal TAS found the same gap size by performing a full resolution convolution (Nikotin *et al.*, 1969 ▸) with a 0.03 meV precision. Thus, this material is a good model example for this work.

Recently, MnF_2_ has attracted renewed interest because it has been classified as an altermagnet (Šmejkal *et al.*, 2022 ▸), supposedly exhibiting spin-dependent band structure symmetries without net magnetization. Previous experimental studies for the altermagnetic splitting of the magnon modes in MnF_2_ were unsuccessful (Morano *et al.*, 2025 ▸), but a recent study revealed the splitting using polarized neutron scattering (McClarty *et al.*, 2025 ▸). We choose to study the antiferromagnetic spin-wave dispersion at the bottom of the dispersion at (*H*00) for *H* = 1 reciprocal-lattice units (r.l.u.). We aim to perform constant-*Q* scans with different resolution tails by varying the instrumental setup.

For the dispersion in MnF_2_ there exists an analytical model (Yamani *et al.*, 2010 ▸) which we have implemented in *McStas* [see Schack *et al.* (2026 ▸) for more details]. We perform simulations of MnF_2_ using a typical doubly focusing cold-neutron TAS model described in Appendix *E*[App appe]. The main component of the *Q* resolution comes from the size (height and width) of the monochromator and the analyser. We add two sets of slits and vary their width equally in order to perform simulations as a function of the incoming and outgoing angular divergence, which effectively varies the *Q* resolution. For each slit setting, we perform a constant-*Q* cut at the gap positions, and for each setting the resolutions are calculated from the covariance matrices given in *McStas*. The methods for calculating such are found in Appendix *F*[App appf] and the resolutions are shown in Table 1 ▸.

We report the three *Q*-resolution components: the in-scattering-plane principal major axis d*Q*_prinmajor_ and principal minor axis d*Q*_prinminor_, and the uncorrelated resolution vertical to the scattering plane d*Q*_vert_. From the table, the two coarse *Q* resolutions d*Q*_prinmajor_ ≃ d*Q*_vert_, which we use as σ_*Q*_ in our analytical function (11). The narrow in-plane resolution d*Q*_prinminor_ varies from half to three-quarters of the size of the other two. It turns out that when the narrow resolution is below around half the size of the other two, the approximations of two broad and one narrow *Q* resolution components hold.

The energy resolution d*E*_atgap_ is the intrinsic instrumental energy resolution at a specific **Q**, namely at the gap position (orange in Fig. 3 ▸). The last column is the projected energy resolution d*E*_proj_, green in Fig. 3 ▸, which almost stays constant over all slit widths. This is to be expected, since we have a Rowland focused (energy focused) monochromator and analyser. The energy resolution d*E*_atgap_ is, as expected, smaller than the projected energy resolution but, opposite to the projected, it increases with increasing slit width. Which energy resolution to use depends on the steepness of the dispersion as illustrated in Fig. 3 ▸.

### 3.1. Fitting to the data

**Figure 4:**
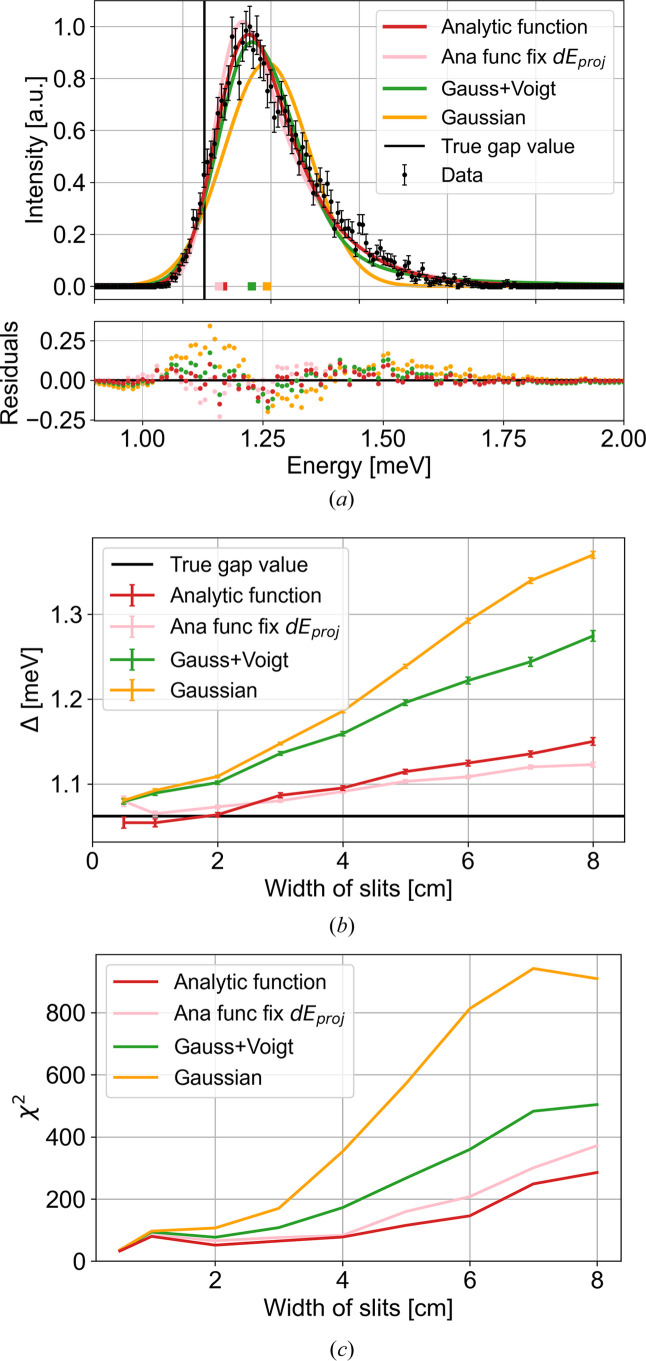
(*a*) Example of simulated constant-*Q* scan through the spin-wave gap in the *McStas* TAS model with a slit of 5 cm. The simulated data are plotted with black error bars and normalized to unity. The fitted peak positions are plotted at zero as coloured squares. Also plotted are the simulated MnF_2_ data with a Gaussian (yellow line), with a combined Gaussian + Voigt function (green line) and with our approximate convoluted gap function (red line). The pink line shows a fit to the convoluted gap function with a fixed σ_*E*_. The fitted gap positions are the coloured squares at zero and the black line shows the nominal gap size at (100), Δ_*T*_ = 1.062 meV. The residuals (data minus fit) are plotted below. The same colour coding is used in plots of (*b*) gap size Δ and (*c*) χ^2^ fits, showing the obtained gap values and χ^2^ values as a function of slit settings, respectively.

The simulated constant-*Q* cuts at increasing slit widths are shown in Fig. 12 in Appendix *G*[App appg]. An example of the 5 cm slit width is shown in Fig. 4 ▸(*a*). Here a clear peak is observed, with a resolution tail towards high energies. In the simulations, no background scattering is simulated. Hence, to prevent small intensity points from being assigned unrealistically small uncertainties, the total intensity error for the fit is defined for all points as the quadrature sum of the simulated error *I*_err_ and a constant term corresponding to 1% of the maximum peak intensity *I*_max_: 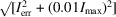. For the fit, we use the resolutions from Table 1 ▸, namely σ_*E*_ = d*E* and 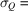

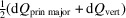
, as the initial parameters. Given the analytical expression for the dispersion reported by Nikotin *et al.* (1969 ▸), the slope of the dispersion is found to be *a* = 15.136 meV Å^−1^. In the fits, the spin-wave velocity *a* is fixed, while all other parameters are allowed to vary within the convoluted gap function in equation (11). Since *a* and σ_*Q*_ are multiplied together, one of them needs to be kept fixed for the fit. For comparison, we also fit with a Gaussian line shape and a combined Gaussian plus Voigt function (see Appendix *B*[App appb]).

### 3.2. The fitted gap sizes

From the three fits to the data from the 5 cm slit setting [Fig. 4 ▸(*a*)], the fitted gap sizes (coloured squares) are 1.239 (2) meV (Gaussian), 1.196 (3) meV (Gaussian plus Voigt) and 1.114 (3) meV (analytical convoluted gap function). From the input parameters to our analytical *McStas* model (Yamani *et al.*, 2010 ▸; Schack *et al.*, 2026 ▸) we obtain a nominal gap size at (100) of Δ_*T*_ = 1.062 meV [horizontal black line in Fig. 4 ▸(*b*)]. All functions overestimate the size of the gap, by 17%, 13% and 5%, respectively. The χ^2^ values, which quantify how well a model describes the data, are, respectively, 572.0, 267.5 and 115.3. It is clear that the Gaussian line shape is not a good fit, while the combined Gaussian + Voigt function and our convoluted gap function seem to describe the data much better. Thus, from the example, our analytical convoluted gap function gives a closer estimate of the gap and describes the data much better than the other two functions.

Generally, looking across various slit widths, the same picture is presented. In Fig. 4 ▸(*b*) the fitted gap sizes for all three fits are plotted as a function of the width of the slits, and Fig. 4 ▸(*c*) shows their respective χ^2^ values. From this, it is clear that our convoluted gap function performs much better than the other two functions. However, as the resolution becomes larger, our function also tends to overestimate the gap size systematically. We strongly recommend that, instead of just looking at the value of χ^2^ as a single number representing the goodness of fit, the user compares the fitted curve with the original data and identifies whether there are any systematic deviations on the low-energy tail, on the high-energy tail, in the central width *etc*. A systematic deviation between the fitted curve and the experimental data can give a systematic error in the extracted gap. We observe a systematic error in the gap determination for all functions. The systematic error in the gap for the analytical function is much smaller than in the case of the Gaussian or Gaussian–Voigt functions, but nevertheless still there.

### 3.3. The fitted resolutions

**Figure 5:**
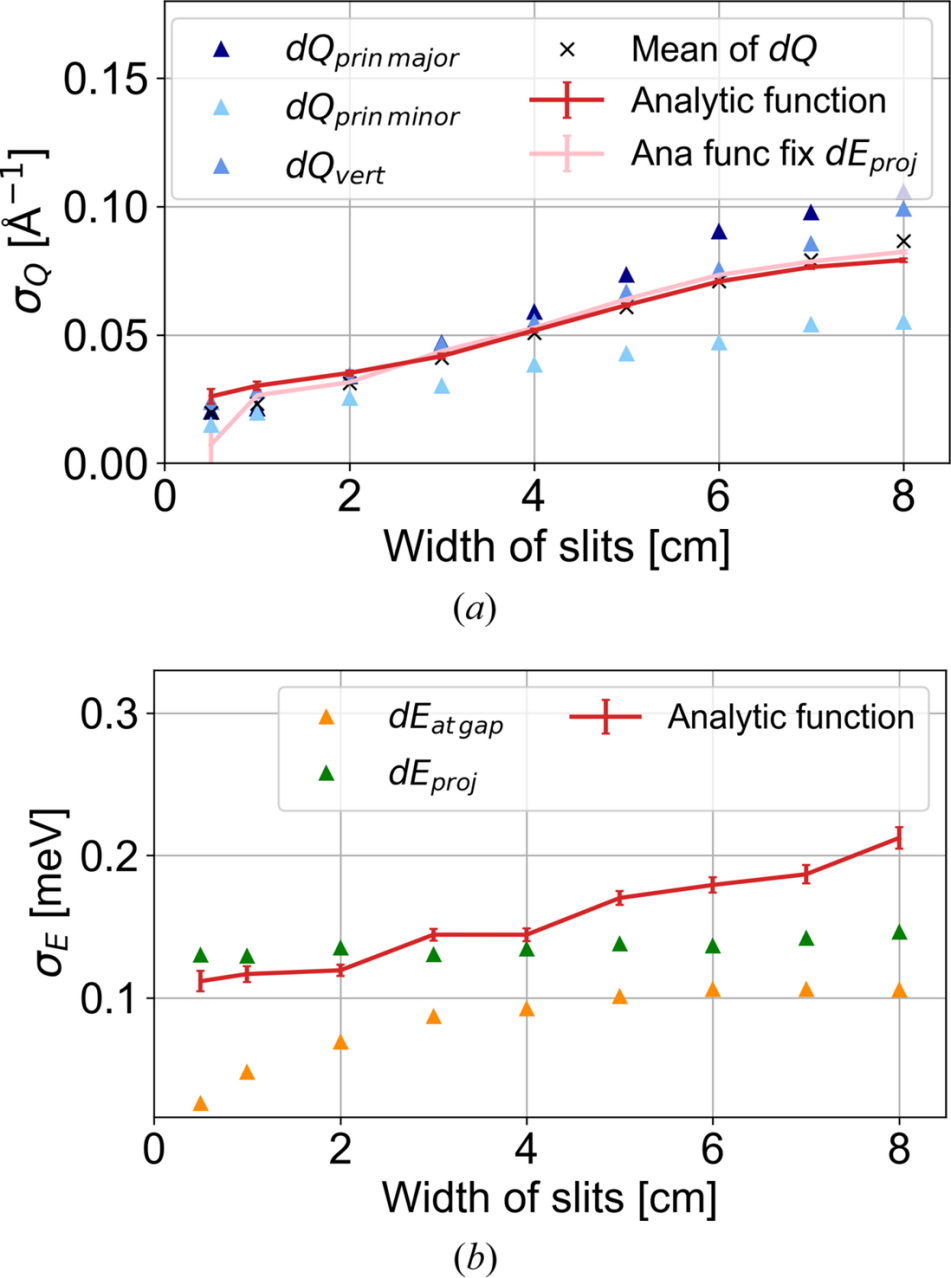
(*a*) Fitted *Q* resolution σ_*Q*_ and (*b*) fitted energy resolution σ_*E*_ from our approximate convoluted gap function equation (11). The red line is where only *a* is fixed in the fit, while the pink line is where both *a* and σ_*E*_ are fixed. The triangles in both figures indicate the respective calculated resolutions from Table 1. The black crosses in panel (*a*) are the three calculated *Q* resolutions averaged. All resolutions are FWHM.

In the example of the 5 cm slits, the simulated resolutions are σ_*Q*_ = 0.0699 Å^−1^ (average of d*Q*_prinmajor_ and d*Q*_vert_) and the energy resolution is either the intrinsic value at the gap σ_*E*_ = 0.101 meV or the projected value of 0.138 meV. Given the steepness of the dispersion and the size of the resolutions, we have the shallow dispersion case (Fig. 3 ▸) and will work with the projected energy resolution. From the convoluted gap function, the fitted parameters are σ_*Q*_ = 0.0615 (7) Å^−1^ and σ_*E*_ = 0.170 (5) meV. Thus, the *Q* resolution is slightly underestimated by the fit, while the energy resolution is overestimated. The general trend for the resolutions fitted with the analytical function [equation (11)] is plotted in Fig. 5 ▸, with the calculated resolutions from Table 1 ▸ plotted as triangles. Fig. 5 ▸(*a*) shows the fitted FWHM *Q* resolution (red error bars) as a function of the slit widths compared with the calculated resolutions from the covariance matrix (blue triangles). One would expect the fitted *Q* resolution to be around the average of d*Q*_prinmajor_ and d*Q*_vert_ (dark- and mid-blue triangles). At small slit widths, we observe the fitted *Q* resolution is slightly overestimated compared with the expected values, while with 3 cm slits it starts becoming slightly underestimated. Taking instead the mean of all three *Q* resolutions (black crosses), we obtain a rather accurate estimate of the fitted *Q* resolution. This is probably because our resolution is not a real pancake, meaning that d*Q*_prinminor_ is not a δ function.

For the fitted FWHM energy resolution in Fig. 5 ▸(*b*) (red error bars), a slight increase is observed with increasing slit width. The intrinsic resolution at the gap, d*E*_atgap_ (orange triangles), is smaller than the fitted resolutions, indicating that our dispersion is shallow (right-hand side of Fig. 3 ▸) such that we need to compare it with the projected energy resolution d*E*_proj_ [green triangles in Fig. 5 ▸(*b*)]. Comparing the fitted energy resolutions and the projected ones, we find that in contrast to the *Q* resolution at small slit widths the fitted resolution is slightly underestimated, while at 3 cm slit width it becomes overestimated. Thus, it seems that the fitting function at large slit widths underestimates the *Q* resolution (12%) and compensates by overestimating the energy resolution (23% compared with the projected energy resolution).

### 3.4. Estimation of the high-energy tail

Since we know the ‘true’ resolutions from Table 1 ▸, we performed fits with a fixed value of the energy resolution, σ_*E*_ = d*E*_proj_. These fits are all plotted in pink in Figs. 4 ▸ and 5 ▸. This procedure gives a gap size slightly closer to the true gap value with only a slightly higher value of χ^2^.

**Figure 6:**
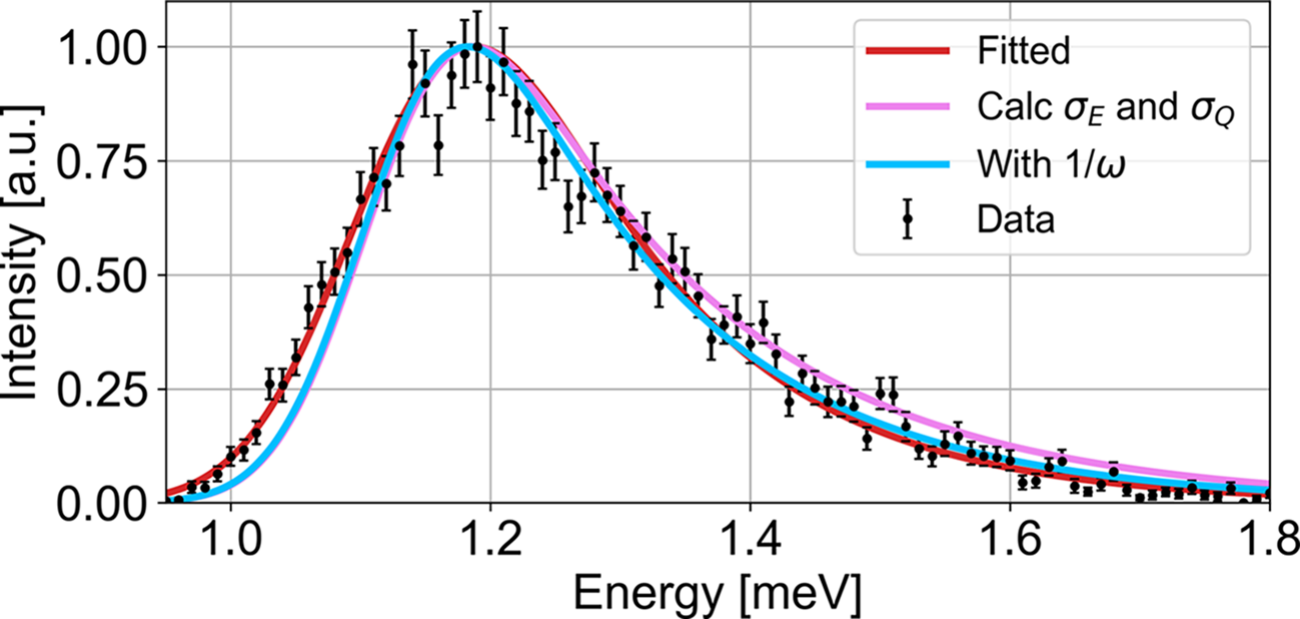
Simulated constant-*Q* scan through the spin-wave gap with a slit of 5 cm. Data are shown with black error bars and the fitted approximate convoluted gap function is plotted in red, as in Fig. 4(*a*). The result of replacing the resolutions by the calculated *McStas* σ_*E*_ and σ_*Q*_ (Table 1 ▸) is plotted in pink. Equation (12) is plotted in blue, and here the calculated *McStas* resolutions are used. The parameters are the same for all plots unless otherwise mentioned.

As noted above, to fit the tail the routine underestimates the *Q* resolution and compensates by overestimating the energy resolution, slightly inflating the extracted gap size. In the 5 cm slit width case, using the calculated rather than fitted values for σ_*Q*_ and σ_*E*_ makes the tail larger (Fig. 6 ▸). Here we present an enlargement of the 5 cm slit scan with the fitted parameters of the analytical convoluted gap function in equation (11) [same as Fig. 4 ▸(*a*)]. To account for this effect, we have to look at the approximations behind our analytical convoluted gap function; the antiferromagnetic magnon dispersion at the gap position can be described by a parabola and the term *S*_*n*_(**Q**) in the structure factor [equation (1)] is constant. If the resolution is large such that we pick up intensity from the sides of the parobola, both approximations will contribute to an overestimation of the tail. To go beyond these assumptions, we include *S*_*n*_(**Q**) ∝ 1/ω in equation (7), such that equation (10) becomes 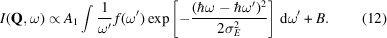
Here *A*_1_ includes all constants. The integral in this expression must be computed numerically, which makes it unstable in fitting procedures. In Fig. 6 ▸ we have plotted equation (12) with the calculated *McStas* resolutions σ_*Q*_ and σ_*E*_. From this, we see that with the 1/ω factor the tail is only slightly reduced, and therefore we deem it unimportant to include this factor. Testing the other approximation, the approximation of the parabola in equation (2), we find deviations when the resolution ellipsoid hits the linear part of the dispersion (Fig. 9 in Appendix *D*1). However, in our fits the error in the gap size is relatively small (5% for the 5 cm slit width). This is probably caused by the fact that the fit chooses a smaller σ_*Q*_ and a larger σ_*E*_ than the correct values. However, in instruments with a broader *Q* resolution, deviations are likely to be more severe.

## 4. Experimental data: MnF_2_

### 4.1. Experimental setup

Experimental data on MnF_2_ were recorded on the TAS-like neutron spectrometer CAMEA at the facility SINQ at the Paul Scherrer Institut (Switzerland). This instrument facilitates a large analyser–detector array in a vacuum tank, giving quasi-continuous coverage along energy transfer and scattering angle. By scanning the sample rotation, one can obtain coverage in two dimensions in *Q* space spanned by two reciprocal-lattice vectors (here [*H*00]–[00*L*]) and in energy (Lass *et al.*, 2023 ▸).

The 3.446 (1) g MnF_2_ sample was positioned in an Orange cryostat and data were acquired at a temperature of 10 K by performing sample rotation scans for five different incoming neutron energies (*E*_i_ = 5, 5.5, 7, 8.5 and 10 meV), each using two different angles of the tank to cover dark angles. The data were converted and analysed using the dedicated software package *MJOLNIR* (Version 1.3.1.post4; Lass *et al.*, 2020 ▸; Lass, 2023 ▸).

### 4.2. Experimental results

**Figure 7:**
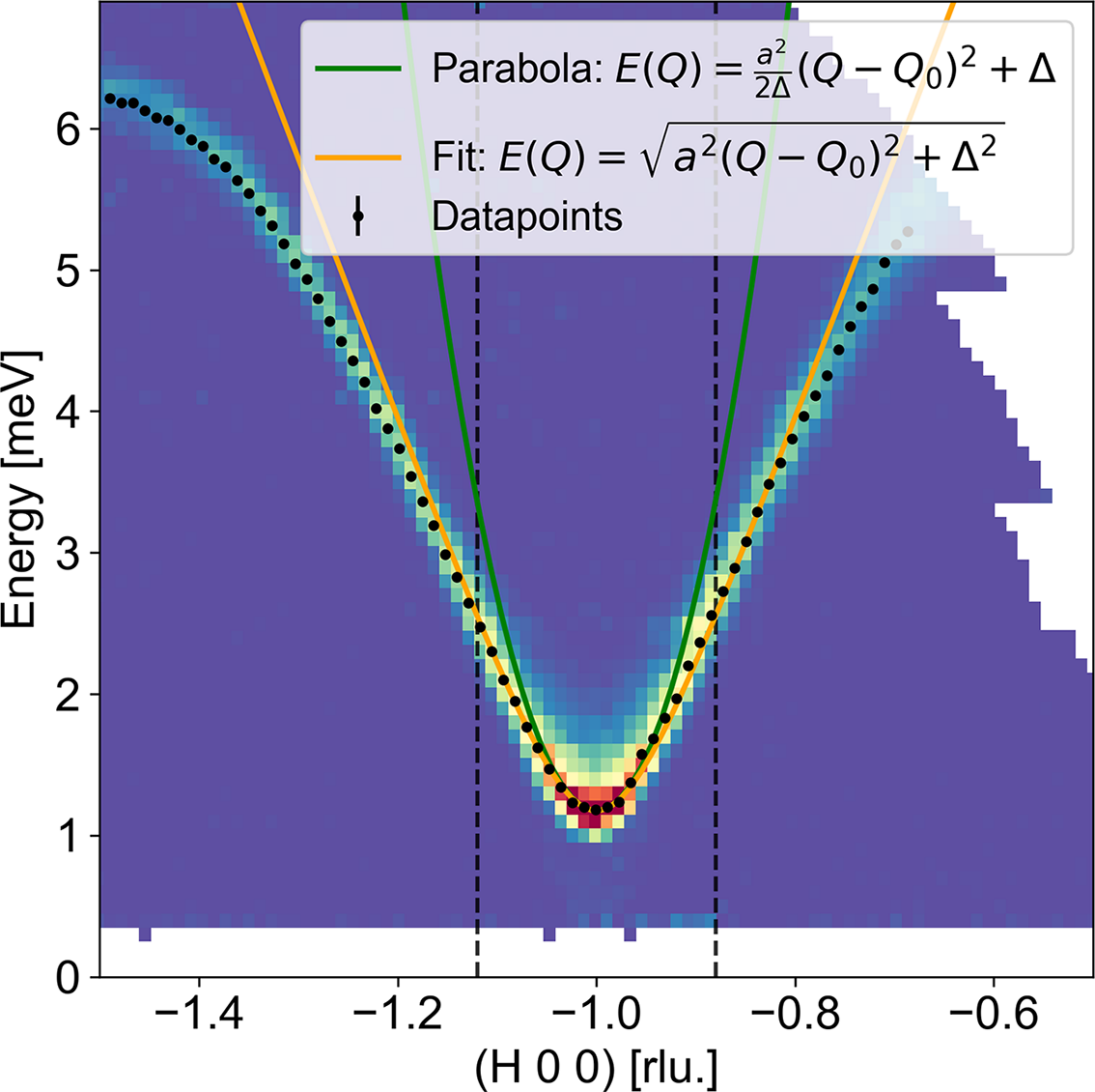
Dispersion of the antiferromagnet MnF_2_ along (*H*00) measured on the TAS-like instrument CAMEA (PSI) at 10 K. The black error bars are constant-*Q* cuts fitted with a Gaussian line shape to find the mode position. Equation (3) is fitted to the error bars in the dashed range (in orange) to find the spin-wave velocity, *a* = 14.89 ± 0.02 meV Å^−1^. The parabola approximation [equation (2)] is plotted in dark green.

**Figure 8:**
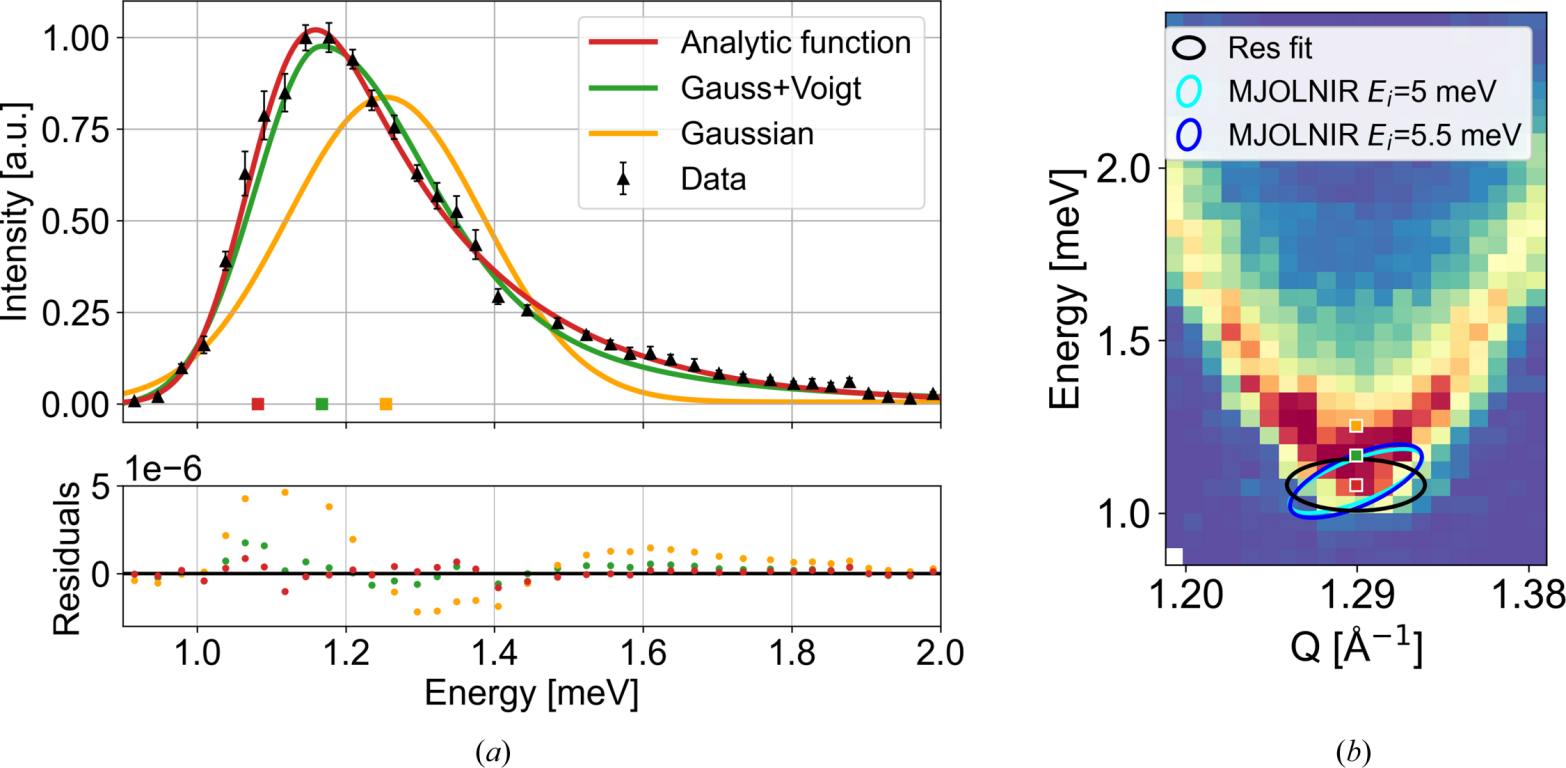
Fitting the gap size of the CAMEA data taken at 10 K with a Gaussian (yellow line), with a combined Gaussian + Voigt in equation (15) (green line) and with our approximate convoluted gap function equation (11) (red line). The fitted gap sizes are plotted as coloured squares. (*a*) Constant *Q* cut at (*H*00), *H* = −1, with an integration width of 0.02 r.l.u. The residuals (data minus fit) are plotted below. (*b*) Enlargement of the dispersion in Fig. 7 with the fitted gap sizes from panel (*a*). The black ellipsis indicates the fitted resolution, and the two blue ellipses are calculated in *MJOLNIR*.

**Figure 9:**
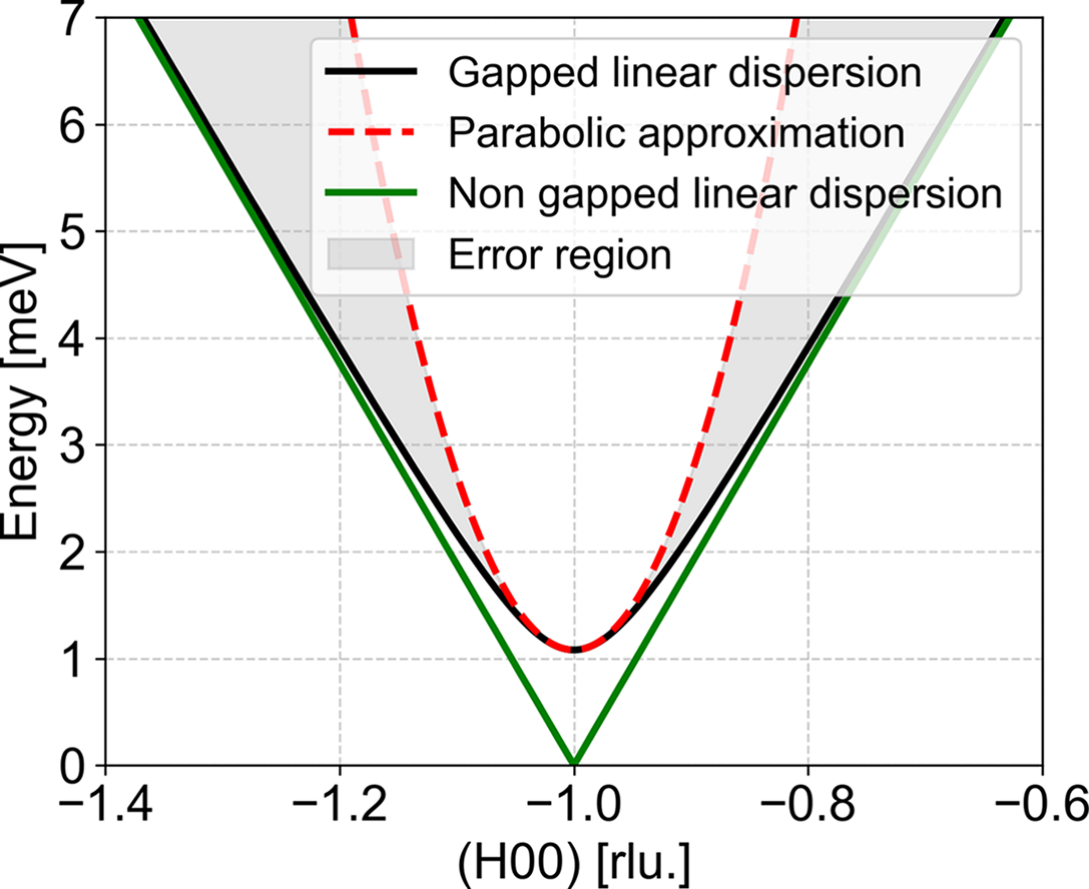
The gapped linear dispersion [equation (3)] (black line) is approximated by a parabola [equation (2)] (dashed red line). The shaded areas indicate the error regions of this approximation. In green, we plot the non-gapped linear dispersion, to show the similarity to the square-root behaviour.

**Figure 12:**
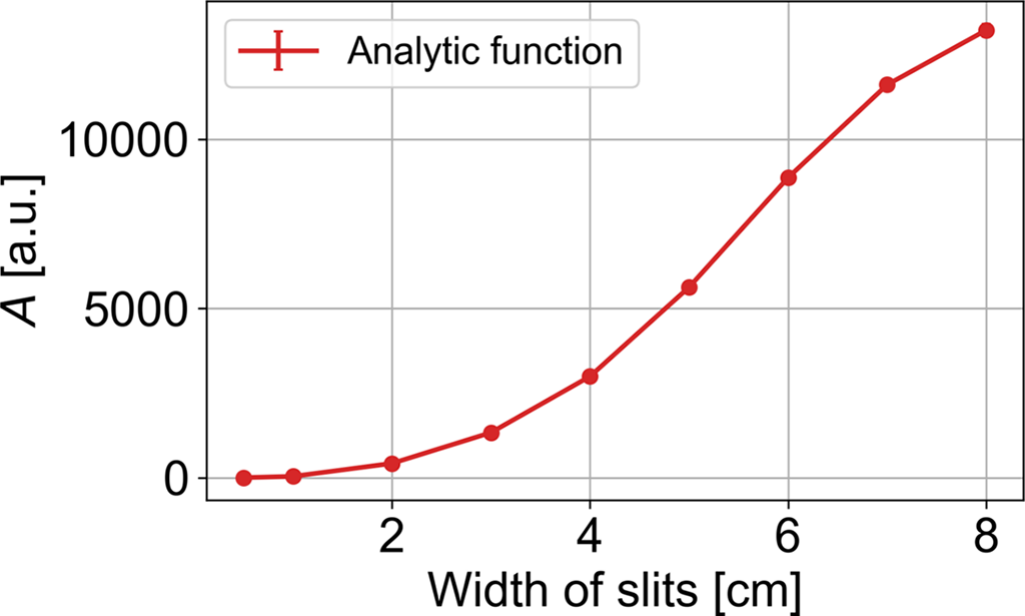
Normalization constant *A* fitted to the *McStas* data using the analytical fitting function in equation (11).

We choose to study the antiferromagnetic spin-wave dispersion along (*H*00) for −1.5 < *H* < −0.5, where the bottom of the dispersion is at *H* = −1 (Fig. 7 ▸). Performing constant-*Q* cuts along *E* and fitting the mode position in each cut with a Gaussian line shape gives the data shown as black error bars. These points are, in turn, used to fit the antiferromagnetic dispersion equation (3) in the linear range (within the dashed vertical lines) to find the slope of the dispersion, *a* = 14.89 ± 0.02 meV Å^−1^. This is very similar to the value found from the analytical expression mentioned in the previous section. The resolutions of the CAMEA instrument are calculated in *MJOLNIR*, which in turn uses the functionality of *Takin* (Weber, 2023 ▸). From this, we obtain the covariance matrix and can in turn calculate the principal axes. CAMEA measures the gap position, 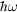 ≃ 1.1 meV, with two incoming energies, *E*_i_ = 5 and 5.5 meV, for which we can calculate the resolution ellipses. The *Q* resolutions (FWHMs) of the principal axes in the scattering plane are the same for *E*_i_ = 5 and 5.5 meV, the major is 0.116 Å^−1^ and the minor is 0.021 Å^−1^. Here, the out-of-scattering plane *Q* resolution (0.095 Å^−1^) is almost the same size as the major principal axis; thus we have two broad and one narrow *Q*-resolution components as prescribed by our approximations. The energy resolution of CAMEA at the gap position has for *E*_i_ = 5 meV an intrinsic energy resolution (FWHM) of 0.087 meV and a projected value of 0.185 meV, while the values for *E*_i_ = 5.5 meV are slightly different, with an intrinsic energy resolution of 0.090 meV and a projected one of 0.213 meV. Both ellipses are plotted in Fig. 8 ▸(*b*). These resolutions are broader than in the simulated TAS instrument above.

### 4.3. Fitting the experimental data

We perform a constant-*Q* cut at (*H*00), *H* = −1, with an integration width of 0.02 r.l.u. in both *Q* directions in the scattering plane [Fig. 8 ▸(*a*)]. For the fit, we keep *a* fixed and all the other parameters are fitted with equation (11). Again, we compare our function with a Gaussian line shape and a Gaussian + Voigt function (Appendix *B*[App appb]). From the figure it is clear that the Gaussian provides a bad fit of the gap data; it finds the gap value to be 1.254 (2) meV, which is highly overestimated. The Gaussian + Voigt function and the convoluted gap function both describe the data much better. The fits yield gap sizes of 1.167 (3) and 1.082 (2) meV, respectively, with χ^2^ values of 502.3 and 200.7, respectively. The fitted gap values are plotted on top of the dispersion in Fig. 8 ▸(*b*). From the excellent agreement with the gap value measured by AFMR, it is clear that our analytical function provides a very accurate means of determining the gap value from the neutron data.

The fitted resolutions are σ_*E*_ = 0.149 (4) meV and σ_*Q*_ = 0.0725 (5) Å^−1^. Compared with the calculated resolutions, σ_*E*_ is slightly underestimated with respect to the projected resolution. On the other hand, σ_*Q*_ is also underestimated compared with the major principal axis. However, the average of the *Q* resolutions in the three directions is 0.0773 Å^−1^, which is very similar to the fitted value, as we also observed from the fits to the simulated data.

One can also decide to fix the energy resolutions to the calculated values. We performed this on our CAMEA data, using both the projected energy resolution and the intrinsic energy resolution at the gap. The fits are shown in Appendix *H*[App apph]. From these fits we obtain gap values of 1.097 (2) and 1.066 (2) meV with χ^2^ values of 277.2 and 395.2, respectively. Thus, fixing the energy resolution to the projected energy resolution (0.185 meV) we get a slightly worse fit, but the fitted gap size becomes larger than the previously reported gap size. Analogously, when fixing the energy resolution to the intrinsic energy resolution at the gap (0.087 meV) we also obtain a non-ideal fit. In the latter case, however, we force the gap to be smaller, since the fit mainly takes the high-energy tail into account. Thus, the obtained fitted energy resolution (0.149 meV) takes a value in between.

## 5. Discussion and conclusion

In this article, we have presented an analytical convoluted gap function, equation (11), which is based on two approximations: (i) the *Q* resolution is coarse in two out of three directions (typical of a focused TAS) and (ii) at the gap, the dispersion follows a parabola. For quadratic excitations, such as ferromagnetic spin waves, the parabola is an exact representation, while for linear excitations, such as antiferromagnetic spin waves, it is merely an approximation. We tested the approximation on antiferromagnetic spin waves in MnF_2_ on simulated data, while effectively varying the *Q* resolution. We also tested it with experimental data from the TAS-like instrument CAMEA in a single experimental setting. In both cases, the convoluted gap function performs much better than a Gaussian line shape or a Gaussian plus Voigt function.

From our simulated data we find that, with increasing *Q* resolution (slit width), the fitting routine tends to underestimate σ_*Q*_ compared with what was expected from the mean of the two coarse *Q* resolutions. Instead, taking the average of the three calculated *Q* resolutions, one finds very good agreement with the fitted *Q* resolution. For the underestimated σ_*Q*_, the fitting routine compensates with increasing σ_*E*_, such that the gap size Δ in effect becomes slightly overestimated. We ascribe this to our parabolic approximation for the bottom of the antiferromagnetic dispersion, which in turn overestimates the high-energy tail above the gap.

We tested our assumption of a constant *S*_*n*_(**Q**) by instead using the more realistic scenario *S*_*n*_(**Q**) ∝ 1/ω. This results in a very slight overestimation of the high-energy tail and a small narrowing of the low-energy part, which might result in a slight reduction of the gap size. However, the resulting function must be evaluated numerically and struggles to converge when implemented in a fitting routine. The overestimation of the high-energy tail is minor, and our analytical function compensates for it by adjusting σ_*Q*_ when this is kept as a free fitting parameter.

For the TAS-like experimental data, the analytical function performs very well, even though CAMEA is a more complex instrument than a standard TAS. Thus, our function has proved in practice to be a good approximation for fitting gaps where the energy resolution is much narrower than the gap value.

When using the analytical function [equation (11)] for fitting, we found the following. Once the parameter *a*, which characterizes the steepness of the dispersion, is fixed according to prior knowledge of the dispersion or from scans away from the minimum, the fitting routine becomes robust with respect to the initial guesses for the remaining parameters. This robustness arises because Δ sets the peak position, σ_*E*_ controls the low-energy width and σ_*Q*_ governs the high-energy tail, with no other parameters influencing these features. The analytical form of the function ensures convergence, whereas fits using a numerically simulated resolution convolution tend to be less stable. Keeping σ_*Q*_ as a fitting parameter allows a first-order correction to the parabolic approximation, hence extending the validity region of the function. In the symmetric line shape limit, the analytical function becomes equivalent to a simple Gaussian, while in the limit of large energy tails the function performs better than any other analytical function. However, if σ_*Q*_ differs significantly from what is expected from the instrument parameters, we recommend performing a full resolution simulation to identify whether the deviation in σ_*Q*_ is due to other effects than resolution convolution. It is only necessary to do something more if the function cannot fit the data. To our knowledge, there is no other analytical function that has a broader range of validity than the one presented here.

Intrinsic line shapes that deviate from sharp dispersions can produce convoluted spectral profiles that are not captured by the analytical function. Examples include strongly damped excitations, which generate low-energy tails, and systems where multi-magnon processes or fractional excitations contribute additional high-energy scattering not accounted for by the resolution function. In cases where the excitations are not sharply dispersive, a separate dedicated analysis is required. Nevertheless, the resolution function presented here provides a means of distinguishing whether the observed spectral features arise from instrumental resolution or from intrinsic physical properties of the sample. Estimating σ_*Q*_ and σ_*E*_ from the experiment (Appendix *A*[App appa]) provides the advantage of distinguishing whether the low-energy width of the peak is limited by instrumental resolution or reflects intrinsic damping in the material. Similarly, it allows one to assess whether the high-energy tail arises solely from resolution effects or is partially due to continuum scattering. To determine whether the high-energy tail contains continuum scattering, the resolution has to be well known. Using the correct resolutions (pink in Fig. 6 ▸), if the data points lie above the analytical function, one can be certain that extra scattering is present and contributes to the high-energy tail. However, if the data lie below the function then a more thorough investigation is needed. Thus, if the objective of an experiment is to quantify continuum scattering, then even if a good agreement between the data and the fit indicates the absence of such scattering, we still recommend a full resolution convolution to exclude any continuum signal.

We find that the parabolic approximation provides an excellent local model at the gap and for small displacements from the minimum, capturing the essential curvature of the linear excitation response with errors that remain below a few percent. However, it should not be extended too far into the wings of the function, where the difference grows rapidly and the true linear excitation departs significantly from the simple quadratic form. The analytical convoluted gap function will essentially always perform better than other analytical functions and can therefore always be used as the initial fitting method. If there are systematic deviations between the fitted curve and the data, then we recommend performing a full resolution convolution to understand whether the deviation is a resolution effect or is caused by additional scattering. As a further development, it is possible to extend the expression to that of a double gap (Lenander *et al.*, 2026 ▸).

To conclude, we have derived an analytical expression for the resolution convoluted line shape of an energy scan through the minimum of a gapped excitation. We have demonstrated, on both simulated and experimental data, that the analytical function provides a good fit to the data. As a result, it provides a significantly better estimate of the gap value than using a Gaussian line shape or a Gaussian plus Voigt function. The analytical function can easily be implemented into fitting routines [an example Python script for fitting is given by Lenander (2026 ▸)] without suffering from difficulties of convergence. The function accounts for the high-energy tail caused by the momentum resolution and, as such, it can be used to identify whether additional continuum scattering is present above the sharp dispersion. Therefore, we recommend the routine use of this analytical function for fitting energy scans through gap minima measured by TAS.

## Figures and Tables

**Figure 10:**
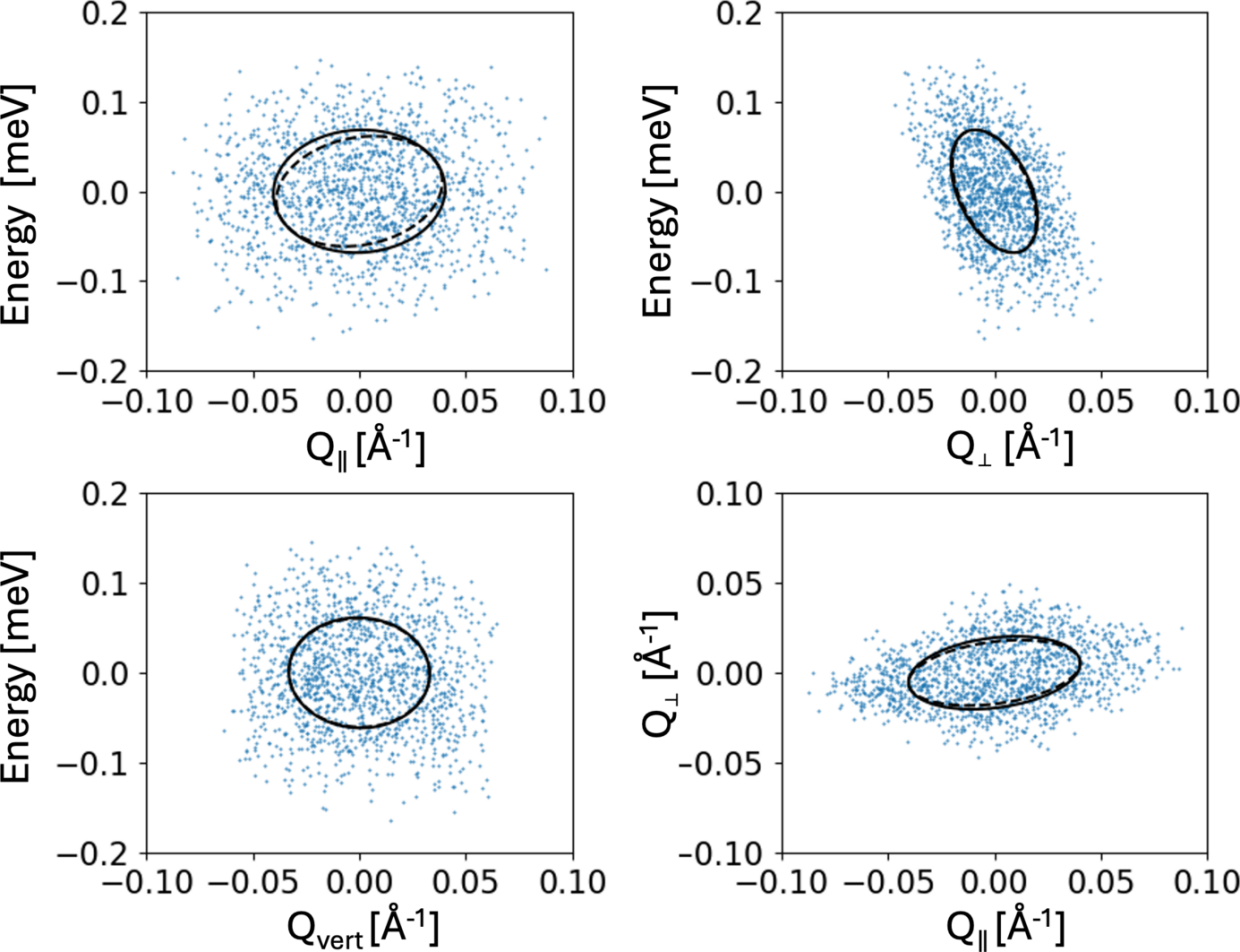
Simulated resolution ellipsoid of the *McStas* TAS model with a slit width of 5 cm. The blue dots show the scattered neutrons, the solid black ellipses are projections of the four-dimensional resolution ellipsoid onto the plotted plane, and the dashed contours mark two-dimensional slices of the full ellipsoid in that plane.

**Figure 11:**
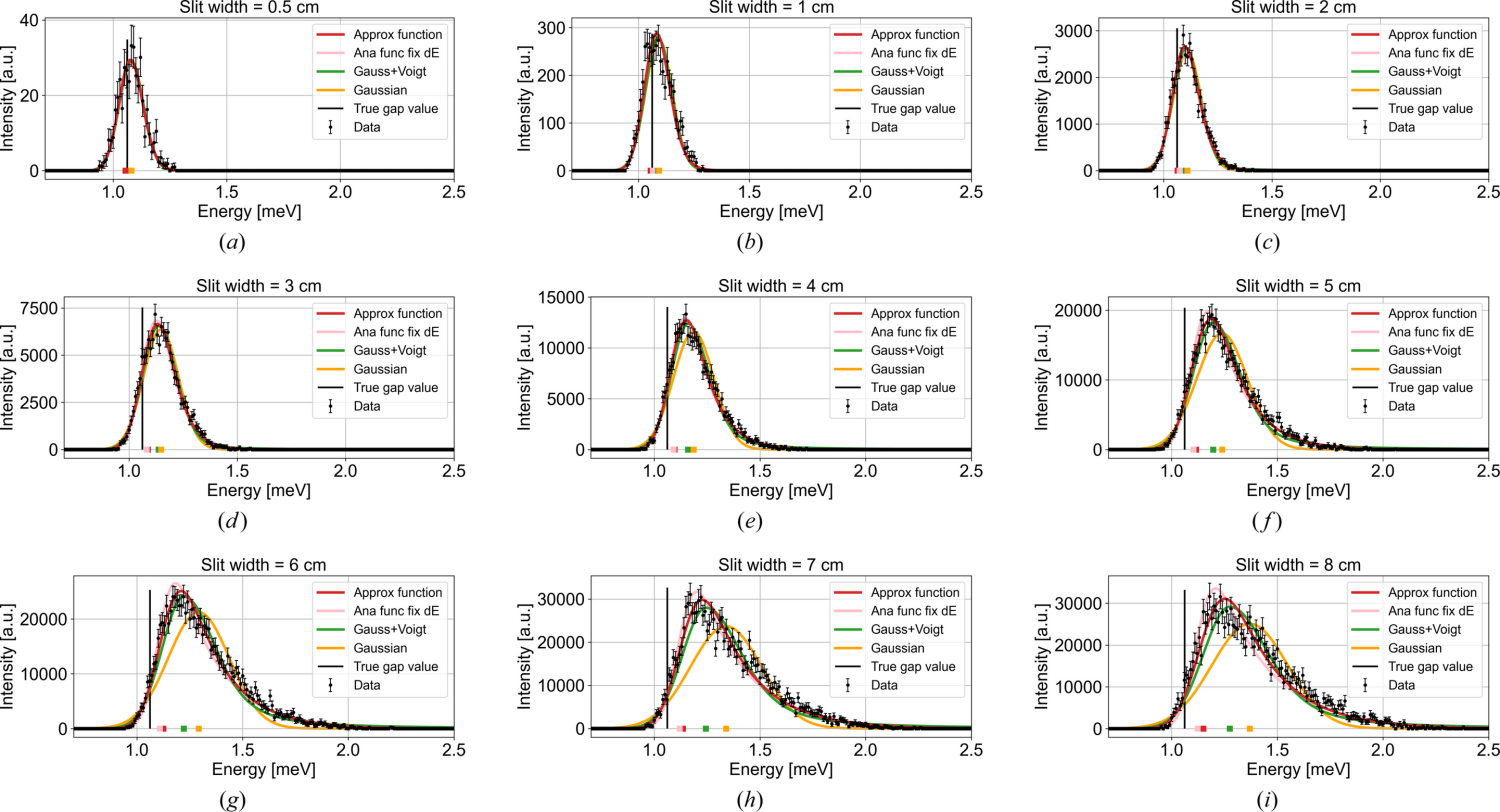
Overview of all simulated constant-*Q* cuts at the gap, varying the slit width from 0.5 to 8 cm, which effectively varies the *Q* resolution. The black error bars are the simulated data and have been fitted with a Gaussian (yellow line), a combined Gaussian plus Voigt in equation (15) (green line) and our approximate convoluted gap function equation (11) (red line). We have also fitted with a fixed value of the energy resolution σ_*E*_, shown in pink. The coloured squares at zero indicate the fitted gap positions.

**Figure 13:**
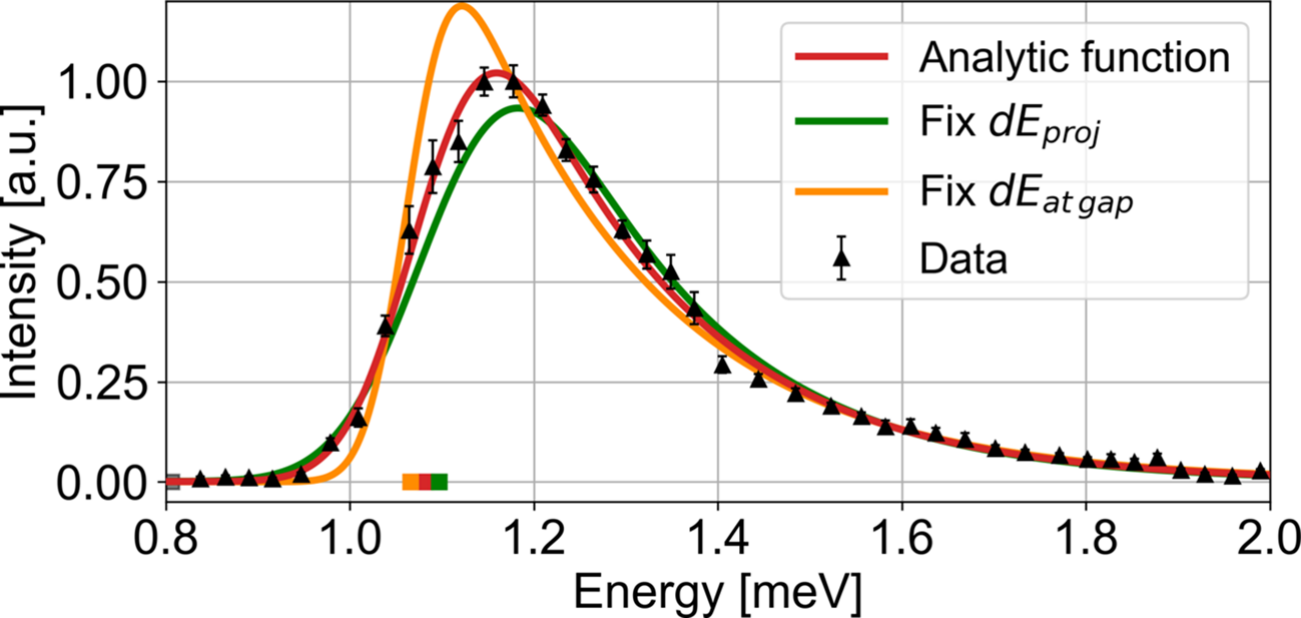
Testing the energy resolution found from the CAMEA data. In red, we fit the data (black error bars) with our approximate convoluted gap function equation (11), keeping σ_*E*_ free. In green, we fix σ_*E*_ to the projected energy resolution, while in orange, we fix σ_*E*_ to the intrinsic energy resolution at the gap.

**Table 1:** Simulated resolution FWHM components as a function of slit width before and after the sample The in-plane principal axes of the *Q* resolution are given by d*Q*_prinmajor_ and d*Q*_prinminor_, while d*Q*_vert_ is the uncorrelated resolution vertical to the scattering plane. The energy resolution d*E*_atgap_ is the intrinsic instrumental energy resolution at a specific **Q**, namely at the gap position (solid orange line in Fig. 3). The last column is the projected energy resolution d*E*_proj_ (solid green line in Fig. 3). The energy resolution is given in meV and the *Q* resolutions are in units of Å^−1^.

Slit width (cm)	d*Q*_prinmajor_	d*Q*_prinminor_	d*Q*_vert_	d*E*_atgap_	d*E*_proj_
0.5	0.0200	0.0148	0.0238	0.0259	0.1300
1.0	0.0213	0.0196	0.0283	0.0479	0.1295
2.0	0.0338	0.0255	0.0343	0.0692	0.1352
3.0	0.0469	0.0301	0.0464	0.0870	0.1344
4.0	0.0589	0.0384	0.0551	0.0925	0.1327
5.0	0.0733	0.0427	0.0665	0.1010	0.1381
6.0	0.0903	0.0470	0.0754	0.1059	0.1365
7.0	0.0976	0.0540	0.0856	0.1061	0.1421
8.0	0.1057	0.0551	0.0990	0.1057	0.1464

## Data Availability

The simulated data and the CAMEA data can be obtained from the authors upon request.

## Appendix A Guide to experimental determination of the parameters in the analytical function

When performing a TAS experiment, one can determine the instrument resolution experimentally by performing a few scans:

(i) The in-plane *Q* resolution d*E*_prinmajor_ and d*E*_prinminor_. One must first perform a crystal rotation scan over the Bragg peak position, and then extend the length of **Q** by 1% and repeat. If the peak intensity is still above half of the central scan, then one should double the step size in the length of **Q** and perform a new crystal rotation scan. From this, the in-plane *Q* resolution ellipsis will be found.

(ii) The out-of-plane *Q* resolution d*E*_vert_. This can be found by tilting the crystal around an in-plane axis perpendicular to the scattering plane and then performing the same type of scan as mentioned above.

(iii) The energy resolution d*E*_proj_ or d*E*_atgap_. Depending on the steepness of the dispersion at the gap compared with the resolution, one needs to use either the projected energy resolution or the intrinsic energy resolution at the gap position (right-hand side of Fig. 3 ▸) in the approximation. For a shallow dispersion one uses the projected energy resolution, which one commonly determines through a scan of an incoherent scatterer, such as vanadium. For a steep dispersion, the intrinsic energy resolution at the gap position is used, and it can be found by performing an energy scan through the Bragg peak.

(iv) The spin-wave velocity *a*. By measuring a constant-energy scan at approximately twice the gap size, one can find the mode positions at the linear part of the dispersion and then use equation (3) to fit *a*.

(v) The curvature of the parabola α. This is the same approach as above, but using equation (2) instead.

Note that this guide finds the *Q* resolution and the energy resolution for elastic scattering, which can vary slightly from the resolution for inelastic scattering. However, it will provide an estimate of the instrumental resolution at the gap, assuming that the gap is close to the elastic line.

## Appendix B Gaussian and Voigt functions

To fit a dispersion to find the mode positions, one will often select a Gaussian line shape, defined as

where *A* is the normalization, μ is the centre of the peak, and σ is the standard deviation or width of the peak.

However, at the bottom of a dispersion, one tends to pick up signal from higher energies, resulting in a tail at energies above the gap. To describe the tail, a Gaussian for the lower-energy part of the peak and a Voigt function for the upper-energy part of the peak can be used. The Voigt profile is a convolution of a Gaussian and a Lorentzian function, typically having a Gaussian peak shape with a Lorentzian tail. The Voigt profile is defined as

where μ is the centre of the peak, σ is the standard deviation of the Gaussian component and γ is the half-width at half-maximum of the Lorentzian component. *w*(*z*) is the Faddeeva function (Dinnebier *et al.*, 2019 ▸) and  denotes the real part. To make the peak asymmetric composed of a Gaussian at lower energies and a Voigt profile at higher energies, the peak is given by

The factor  ensures continuity at *x* = μ by scaling the Voigt profile to match the Gaussian at the peak centre.

## Appendix C Derivation of the convoluted gap function

### c1. Numerical derivation

Using the approximations presented in the main text, namely equations (6) and (7), we can write the intensity given by equation (4) as

First, to simplify matters, we assume the gapped dispersion to be centred around zero, such that **Q**_0_ = **0**. We use polar coordinates to rewrite  = *Q*^2^ and d**Q**′ = *Q* d*Q* dθ:

Substituting *Q*^2^ =  we get d*Q* = (2*Q*α)^−1^dω, which is used to simplify the expression:

Importantly, we only start picking up intensity when we reach the gap position, , so we use the Heaviside step *H*:

The intensity becomes an asymmetric function *f*(ω) that is being convoluted with the energy resolution. We add the Gaussian normalization constants and experimental data, and all the other constants, *e.g.* the prefactor of *S*(**Q**, ω) and the intensity normalization of the instrument, are described by a normalization constant *A*. To account for the instrument background, the scalar *B* is used. Thus, the function for fitting a gapped dispersion becomes

However, this is a function to be determined numerically, which makes it more difficult in practice for a fitting routine to converge at the global minimum. Thus, we are interested in finding an analytical expression.

### c2. The analytical derivation

We define the integral in equation (20) by *J*:

where  and the Heavyside step function makes the integration range  ∈ [Δ, ∞]. By completing the square, we can rewrite the exponent as

We define  and add and subtract μ^2^ such that the exponent becomes

Hence,

Using the standard Gaussian tail identity,

and we find

Substituting back  gives the analytical expression

This form is valid for all . The Heaviside function in the base, *f*(ω) in equation (19), ensures support only for  ≥ Δ, but the convolution itself yields a smooth non-zero tail for all  given by the erfc. Comparing the numerical expression in equation (20) and the analytical function in equation (28) gives an agreement better than 0.01%; this tiny difference we ascribe to the finite grid resolution in the numerical integral.

## Appendix D Limitations of the convoluted gap function

Our convoluted gap model has some limitations as to when the expression is valid. The assumptions used have to be fulfilled to obtain the best possible fit.

### d1. Accuracy and limits of the parabolic approximation

Our approximated convoluted gap function in equation (11) will overestimate the tail above the gap position due to the approximation of the linear excitation in equation (3) following the shape of the parabola in equation (2). In Fig. 9 ▸ we illustrate the error regions of the approximation, as well as the non-gapped linear dispersion.

Physically, the parabolic approximation is reliable for describing the near-gap region, where the dispersion has a constant curvature, but not for the outer regions where the dispersion becomes more linear due to the square-root dependence. In those tails, the approximation exaggerates the dispersion value and leads to an overprediction of the total signal when integrated. The degree to which this overestimation is apparent depends on the instrumental resolution. The overestimation can be mitigated by allowing σ_*Q*_ to be optimized in the fitting process.

In the case of a focused TAS, the instrumental resolution is typically anisotropic, with two wide components and one narrow one in momentum transfer. This geometry produces a resolution ellipsoid that is elongated along certain reciprocal-space directions, effectively averaging the scattering intensity over a broad region in *Q* while maintaining sharp definition along one axis. As a result, the measured dispersion appears smoother and more parabolic, since the averaging suppresses the intrinsic square-root flattening of the linear dispersion. The broad *Q* acceptance leads to a systematic overestimation of the intensity in the high-energy tails, while the narrow component preserves an accurate determination of the gap position. Thus, in focused TAS measurements, the parabolic approximation often provides an excellent apparent fit near the gap.

### d2. Isotropic spin-wave velocity

We assume an isotropic spin-wave velocity *a*. If the real material has anisotropic spin-wave velocities *a*_*i*_ along different crystal axes, then

The small-*Q* expansion then yields an anisotropic quadratic form , *i.e.* elliptical constant-energy contours instead of circular. Thus, the isotropic parabola is only valid when the anisotropy is small.

### d3. The Bose factor

Thermal fluctuations can affect the spin-wave spectrum, which is described in the thermal population factor (the Bose factor)  = , where *k*_B_ is the Boltzmann constant and *T* is the temperature. We assume a dispersion spectrum at low temperatures, where thermal fluctuations do not affect the spectrum. The Bose factor at low temperatures is , where *n*(ω) + 1 ≃ 1, and we get equation (11).

In the high-temperature (classical) limit, the Bose factor becomes approximately . This factor reflects the occupation of excited states rather than the underlying spin dynamics. Thus, at high-temperatures the factor  is added to the integral in equation (10), which cannot be written in an analytical form. However, instead of having *J* in equation (21) we can define *K* as

## Appendix E *McStas* model

For the dispersion in MnF_2_, we implemented an analytical model of Yamani *et al.* (2010 ▸) in *McStas* (Schack *et al.*, 2026 ▸). We simulated data from MnF_2_ in *McStas*. To simulate the spectrometer, we used a doubly focusing cold-neutron TAS model with:

(i) A 15 × 15 cm source.

(ii) A 2 × 2 cm slit before the monochromator as a virtual source.

(iii) A curved 20 × 15 cm pyrolitic graphite (PG) monochromator with a horizontal curvature of 2.7 m and a vertical curvature of 0.9 m. This was positioned 1.5 m before the sample.

(iv) A slit with a variable width to control the resolution, 0.9 m before the sample.

(v) A cylindrical sample, 1.25 cm tall and 1 cm in diameter, representing antiferromagnetic spin waves from MnF_2_. We here used the new SpinWave_BCO component in *McStas* (Schack *et al.*, 2026 ▸).

(vi) A second slit with the same variable width, 0.9 m after the sample.

(vii) A 15 × 15 cm PG analyser with a horizontal curvature of 1.67 m and no vertical curvature. The analyser was positioned 1 m from the sample and was set to reflect at a constant *E*_f_ = 5.0 meV.

(viii) A single energy-sensitive detector, 2.5 × 1.25 cm.

In the simulations, the source energy range is limited to avoid higher-order scattering from the monochromator. Likewise, the detected energy range is limited to avoid higher-order scattering from the analyser.

We simulated the resolution function of the instrument by replacing the sample and detector with the *McStas* components Res_sample and Res_Monitor, respectively. The method is described by Lefmann *et al.* (2000 ▸). The covariance matrices were calculated using the *McStas* resolution calculator program *mcresplot*. The instrumental resolutions at nine different slit widths are reported in Table 1 ▸. As an example, the resolution ellipses for the setting with slit widths of 5 cm are presented in Fig. 10 ▸, showing four different projected resolutions.

## Appendix F Calculating the resolutions from the covariance matrix

The instrumental resolution of a neutron spectrometer can be fully described by a covariance matrix **C** which characterizes the uncertainties and correlations between the four measured quantities: the three components of the momentum transfer **Q**, and the energy transfer *E*.

In the laboratory (Cartesian) coordinate system, the resolution covariance matrix is written as

Each element 〈Δ*A*Δ*B*〉 represents the covariance between parameters *A* and *B*. The diagonal terms give the variance (square of 1σ uncertainty) of *Q*_*x*_, *Q*_*y*_, *Q*_*z*_ and *E*, while the off-diagonal terms quantify how uncertainties in these quantities are correlated due to the geometry and kinematics of the instrument.

### f1. Coordinate transformations

Although **C** is often expressed in the laboratory coordinate system (*Q*_*x*_, *Q*_*y*_, *Q*_*z*_, *E*), it is frequently more useful to represent it in a local coordinate frame defined relative to the measured scattering geometry, such as (*Q*_∥_, *Q*_⊥_, *Q*_vert_, *E*). Here,

(i) *Q*_∥_ is parallel to the nominal **Q** direction.

(ii) *Q*_⊥_ lies in the scattering plane but perpendicular to *Q*_∥_.

(iii) *Q*_vert_ is perpendicular to the scattering plane.

The transformation from the laboratory frame to the local frame is given by

where **R** is a rotation matrix whose columns define the local basis vectors expressed in laboratory coordinates. This transformation rotates the covariance matrix into a frame that is physically aligned with the measurement direction.

Expressing the covariance matrix in (*Q*_∥_, *Q*_⊥_, *Q*_vert_, *E*) provides a more intuitive picture of the instrumental resolution in reciprocal space and energy. In this frame, the principal axes of the resolution ellipsoid typically align more closely with physically meaningful directions – such as along the dispersion relation or perpendicular to the scattering plane – making it easier to visualize how instrumental uncertainties contribute to the measured signal.

### f2. Determination of the principal axes of the *Q* resolution in the scattering plane

To visualize the resolution in the scattering plane, *e.g.* (*Q*_*x*_, *Q*_*y*_), the full covariance matrix is projected using a projection matrix **P**, defined such that

where **P** selects the desired subspace [*e.g.* the (*Q*_*x*_, *Q*_*y*_) plane].

The principal axes of the resolution ellipse in this plane are obtained by diagonalizing the projected covariance matrix,

where λ_*i*_ and **v**_*i*_ are the eigenvalues and eigenvectors, respectively. Each eigenvector **v**_*i*_ defines the orientation of a principal axis of the ellipse, while the corresponding eigenvalue λ_*i*_ gives the variance along that direction.

The 1σ widths along the principal axes are

and the full-width at half-maximum (FWHM) values are

These are the values we report as the resolution of the instrument.

### f3. Energy resolution from the covariance matrix

The instrumental covariance matrix in equation (31) can be rewritten such that the momentum transfer is written as **Q** = (*Q*_*x*_, *Q*_*y*_, *Q*_*z*_):

where **M**_*QQ*_ is a 3 × 3 covariance submatrix in momentum space,  contains the cross-covariances between **Q** and *E*, and *M*_*EE*_ is the variance of the energy transfer.

Two different definitions of the energy resolution can be derived from the covariance matrix **M**, depending on whether the momentum **Q** is considered to be fixed or not. Whether one needs to use one or the other depends on the steepness of the dispersion and the size of the *Q* resolution (right-hand side of Fig. 3 ▸). The intrinsic energy resolution at a specific *Q* (orange in Fig. 3 ▸), σ_*E*|**Q**_, is typically smaller than the projected resolution (green in Fig. 3 ▸), σ_*E*_, because fixing **Q** removes the influence of the **Q**–*E* correlations that tilt the resolution ellipsoid in (**Q**, *E*) space.

#### f3.1. Intrinsic energy resolution at specific *Q*

If the momentum transfer **Q** is fixed, the intrinsic energy resolution variance is obtained from the conditional variance of *E* given **Q**,

The corresponding one-standard-deviation width and the FWHM are

This represents the intrinsic energy broadening of the instrument, *i.e.* the spread in *E* when we are at a specific **Q**, like at the gap.

#### f3.2. Projected energy resolution.

If the momentum transfer is not fixed, the total projected energy resolution variance is simply the marginal variance of *E*,

with

This quantity includes both the intrinsic energy broadening and the additional contribution from the correlations between **Q** and *E*. It therefore represents the overall apparent energy resolution observed when the measurement integrates over the finite **Q** range the *Q* resolution includes.

## Appendix G Fitting the simulated data

As mentioned in the main text, we have simulated constant-*Q* cuts at the gap with varying slit widths, which effectively varies the *Q* resolution. All simulated results have been fitted with our analytical function (11), a Gaussian line shape and a combined Gaussian plus Voigt function (Appendix *B*[App appb]). All the fits can be seen in Fig. 11 ▸; these are not normalized. The fitted parameters are plotted in earlier figures: the gap size [Fig. 4 ▸(*b*)], the *Q* resolution and energy resolution (Fig. 5 ▸), and the χ^2^ value [Fig. 4 ▸(*c*)]. Additionally, we fitted the normalization constant *A*, which can be seen in Fig. 12 ▸. Noting the value of *A* as a function of slit width, it is clear that the larger the slit width, the more neutrons in the detector, up to a saturation value at approximately 8 cm slit width.

## Appendix H Fixed energy resolution in fitting the CAMEA data

We fit our approximate convoluted gap function [equation (11)] to the experimental CAMEA data in Fig. 13 ▸, with the spin-wave velocity *a* as the only fixed parameter. From the fit, we get a gap size of Δ = 1.082 (2) meV, a *Q* resolution σ_*Q*_ = 0.0308 (2) Å^−1^, an energy resolution σ_*E*_ = 0.063 (2) meV and χ^2^ = 200.7. Fixing the energy resolution to the calculated resolution, σ_*E*proj_ = 0.0786 (8) meV and σ_*E*atgap_ = 0.0369 (4) meV, we fitted the gaps once again. For the projected energy resolution, we get an overestimated gap size of 1.097 (2) meV and a slightly larger χ^2^ = 277.2. For the intrinsic energy resolution at the gap, we get a smaller gap size of 1.066 (2) meV with a much larger χ^2^ = 395.2. The energy resolution found by the fitting function lies between the calculated projected and intrinsic resolution values at the gap. This shows us that the ‘effective’ *Q* resolution lies between our two extreme cases and that the choice of energy resolution influences the gap size obtained from the fit.
